# GPER1 signaling restricts macrophage proliferation and accumulation in human hepatocellular carcinoma

**DOI:** 10.3389/fimmu.2024.1481972

**Published:** 2024-11-08

**Authors:** Yanyan Yang, Yongchun Wang, Hao Zou, Zhixiong Li, Weibai Chen, Zhijie Huang, Yulan Weng, Xingjuan Yu, Jing Xu, Limin Zheng

**Affiliations:** ^1^ MOE Key Laboratory of Gene Function and Regulation, School of Life Sciences, Sun Yat-sen University, Guangzhou, China; ^2^ State Key Laboratory of Oncology in South China, Guangdong Provincial Clinical Research Center for Cancer, Sun Yat-sen University Cancer Center, Guangzhou, China

**Keywords:** macrophages, GPER1, proliferation, hepatocellular carcinoma, tumor microenvironment

## Abstract

**Background:**

Sex hormones and their related receptors have been reported to impact the development and progression of tumors. However, their influence on the composition and function of the tumor microenvironment is not well understood. We aimed to investigate the influence of sex disparities on the proliferation and accumulation of macrophages, one of the major components of the tumor microenvironment, in hepatocellular carcinoma (HCC).

**Methods:**

Immunohistochemistry was applied to assess the density of immune cells in HCC tissues. The role of sex hormone related signaling in macrophage proliferation was determined by immunofluorescence and flow cytometry. The underlying regulatory mechanisms were examined with both *in vitro* experiments and murine HCC models.

**Results:**

We found higher levels of macrophage proliferation and density in tumor tissues from male patients compared to females. The expression of G protein–coupled estrogen receptor 1 (GPER1), a non-classical estrogen receptor, was significantly decreased in proliferating macrophages, and was inversely correlated with macrophage proliferation in HCC tumors. Activation of GPER1 signaling with a selective agonist G-1 suppressed macrophage proliferation by downregulating the MEK/ERK pathway. Additionally, G-1 treatment reduced PD-L1 expression on macrophages and delayed tumor growth in mice. Moreover, patients with a higher percentage of GPER1^+^ macrophages exhibited longer overall survival and recurrence-free survival compared to those with a lower level.

**Conclusions:**

These findings reveal a novel role of GPER1 signaling in regulating macrophage proliferation and function in HCC tumors and may offer a potential strategy for designing therapies based on understanding sex-related disparities of patients.

## 1 Introduction

Hepatocellular carcinoma (HCC) is one of the most prevalent tumors and is associated with increasing mortality worldwide, posing a significant threat to human health (1). Epidemiological observations have reported sex disparities in the incidence and progression of HCC (1–3). Generally, males have a higher risk of HCC and a worse prognosis than females. However, the mechanisms underlying these differences are not fully understood.

Evidence has suggested that distinct immune responses in males and females contribute to variations in the incidence and efficacy of treatment for infections, autoimmune diseases and cancers (4). For instance, females generally display a stronger response to pathogens (5), have greater vaccine efficacy (6) and are more susceptible to autoimmune diseases compared to males (7, 8). In the context of tumors, there have been reports of sex disparities in the functions of tumor-infiltrating immune cells mediated by sex hormone signaling in melanoma and colorectal cancer (9–11). Macrophages constitute a major component of the leukocyte infiltrate in HCC. Educated by signals in the tumor microenvironment, macrophages undergo polarization and acquire a phenotype that promotes tumor growth (12, 13). Our previous study has demonstrated that self-replication serves as an important mechanism for macrophage accumulation, and that proliferating macrophages exhibit an immunosuppressive phenotype in HCC tumor tissues (14). However, the influence of sex disparities on the proliferation and accumulation of macrophages in HCC remains unclear.

Sex hormones and their receptors play a crucial role in mediating the differences between males and females, both in normal physiological conditions and in pathological situations. G protein–coupled estrogen receptor 1 (GPER1), a non-classical estrogen receptor, is found in various cell types and is involved in regulating a wide range of physiological and pathological responses (15–17). For instance, GPER1 is essential in protecting fetal health from maternal inflammation caused by pathogen infections through suppressing IFN signaling in fetal tissues (16). Additionally, the activation of GPER1 signaling has been shown to regulate the proliferation of tumor cells in various types of cancer (18, 19). However, the role of GPER1 in regulating macrophage proliferation remains unclear.

In this study, we discovered that the accumulation and proliferation level of macrophages in tumor tissues of HCC were significantly higher in male patients compared to females. Our mechanical study demonstrated that the activation of GPER1 signaling restrained macrophage proliferation and accumulation. It also led to a decrease in PD-L1 expression on macrophages and a delay in tumor growth in mice. Moreover, patients with a higher percentage of GPER1^+^ macrophages exhibited longer overall survival and recurrence-free survival compared to those with a lower level. These findings reveal a novel role of GPER1 signaling in the tumor microenvironment that regulates macrophage proliferation and function in HCC.

## 2 Materials and methods

### 2.1 Patients and specimens

Liver tissue samples were obtained from patients with pathologically confirmed HCC who had not received any anticancer therapy prior to sampling. Individuals with a concurrent autoimmune disease, HIV, or syphilis were excluded. Samples from 48 HCC patients who had complete follow-up data were included to assess overall survival (OS) and recurrence-free survival (RFS) using immunofluorescence staining of GPER1 and CD68. OS was defined as the time between surgery and either death or the last observation for patients who survived. RFS was defined as the time between surgery and either the first recurrence or death, or the last observation for patients without recurrence. The clinical characteristics of these patients are summarized in **Supplementary Table S1**. In addition, fresh biopsy specimens from 7 HCC patients were used to isolate tumor-infiltrating leukocytes for flow cytometry analysis. All samples from HCC patients were coded anonymously in accordance with local ethical guidelines (as stipulated by the Declaration of Helsinki). Written informed consent was obtained from all participants prior to study onset. The use of human subjects for this study was approved by the Institutional Review Board of Sun Yat-sen University Cancer Center.

### 2.2 Culture of HCC cell lines and preparation of tumor culture supernatants

The hepatoma cell lines (HepG2, Huh7, SK-Hep-1and Hepa1-6) were obtained from the American Type Culture Collection (ATCC). All cells were regularly tested for mycoplasma contamination using the single-step polymerase chain reaction (PCR) method. The cells were cultured in DMEM (Gibco) supplemented with 10% FBS (Gibco), 100 U/mL penicillin (Sigma-Aldrich Corp), and 100 µg/mL streptomycin (Sigma-Aldrich Corp) in a humidified 5% CO2 incubator at 37°C. To prepare tumor culture supernatants (TSN), 5 × 10^6^ tumor cells were plated in 10 ml complete medium in 10-cm dishes for 24 hours. Then, the medium was changed to phenol red and serum-free DMEM. After 48 hours, the supernatant was collected, centrifuged, and stored in aliquots at -80°C (14).

### 2.3 Monocyte purification from human peripheral blood and macrophage generation

Human monocytes were isolated as previously described (20, 21). Briefly, peripheral blood mononuclear cells (PBMCs) were isolated from the buffy coats derived from healthy donors’ blood by Ficoll density gradient centrifugation. CD14^+^ monocytes were then purified from PBMCs using magnetic beads (Miltenyi Biotec) according to the manufacturer’s instructions. The purified monocytes were cultured in DMEM supplemented with 10% human AB serum for 7 days to generate macrophages. Afterward, the culture medium was replaced with phenol red-free DMEM (Procell) containing 2% AB serum and 20% TSN to mimic a relatively nutrient-deficient tumor microenvironment. Meanwhile, in certain experiments as indicated, macrophages were treated with biochemical reagents, including G-1 (10008933, Cayman), G-15 (14673, Cayman) or 17β-estradiol (E8875, Sigma-Aldrich).

### 2.4 Mouse tumor models and treatments

All animal experiments were approved by the Institutional Animal Care and Use Committee of Sun Yat-sen University (Guangzhou, China). Male C57BL/6J mice were purchased from the Guangdong Medical Laboratory Animal Center and maintained under specific pathogen-free conditions. The mice used in the experiments were between 6 and 8 weeks old. For the subcutaneous tumor model, a total of 1×10^6^ Hepa1-6 cells were subcutaneously transplanted into the flanks of mice. For the orthotopic tumor model, a total of 1×10^6^ Hepa1-6 cells were suspended in 25 µl of 50% basement membrane extract (354234, corning) and injected into the left lobe of the liver of anesthetized mice. The mice began receiving daily subcutaneous injections of G1 (4mg/kg) dissolved in a solvent containing 10% DMSO (MP), 30% PEG300 (TargetMol), 5% Tween 80 and 55% ddH2O on day 4 (orthotopic tumor model) or day 5 (subcutaneous tumor model). The control mice received a 200 µl dose of the solvent.

### 2.5 Immunohistochemistry

Formalin-fixed and paraffin-embedded HCC samples were cut into 4-μm sections and processed for IHC as previously described (14). Following incubation with anti-human CD3 (MA514524, Thermo Fisher Scientific), anti-human CD20 (ab78237, Abcam), anti-human CD15 (ZM-0037, ZS), anti-human CD68 (ZM-0060, ZS), anti-human CD163 (ab182422, Abcam), anti-human CD204 (KAL-KT022) or anti-mouse F4/80 (70076S, CST) antibodies (Abs), the sections were then stained with the corresponding secondary Abs and visualized using 3,3’-diaminobenzidine (Nichirei). An automatic slide scanner (KF-PRO-020) was used to scan the sections, and then positive cells were quantified by HALO image analysis software (Indica Labs).

### 2.6 Immunofluorescence staining

Formalin-fixed and paraffin-embedded HCC sections were processed as described previously (14). For human tumor sections, anti-human CD68, anti-human Ki67 (ZM-0167, ZS) and anti-human GPER1 (PA5-109319, Thermo Fisher Scientific) Abs were used. For mouse tumor sections, anti-mouse F4/80 (700767, CST) and anti-mouse Ki67 (ab15580, Abcam) Abs were used. Immunofluorescence signals were amplified by a tyramide signal amplification kit (PANOVUE) as instructed by the manufacturer for visualization. Sections were scanned using the Polaris Fully Automatic Digital Slide Scanner (Akoya Biosciences), and then positive cells were quantified using HALO image analysis software (Indica Labs).

For immunofluorescence staining of cultured cells, the cells growing on coverslips were fixed with tissue/cell fixation buffer for 15 minutes at room temperature. Afterward, they were rinsed with PBS and permeabilized and blocked with PBS containing 5% BSA and 0.3% Triton X-100 for 1 hour at room temperature. The cells were then incubated overnight at 4°C with the primary antibody against human Ki67. This was followed by exposure to Alexa Flour 488-conjugated anti-mouse IgG. For double staining of Ki67 and GPER1 or ERα, the cells were simultaneously incubated with primary Abs against human Ki67 and GPER1 (PA5-109319, Thermo Fisher Scientific), or ERα (ab16660, Abcam). They were then exposed to Alexa Flour 488-conjugated anti-rabbit IgG and Alexa Flour 555-conjugated anti-mouse IgG. Nuclei were counterstained with DAPI. The immunofluorescence staining images were visualized using a high-resolution confocal laser microscope (LSM880 with fast airyscan, Zeiss).

### 2.7 EdU incorporation assay

Human monocyte-derived macrophages were either left untreated or treated with 20% Huh7-TSN for 48 hours, in the presence or absence of G-1 (1 μM), and then the cells were cultured with EdU (5-ethynyl-2’-deoxyuridine) at a final concentration of 1 μM for 5 hours. Afterward, the cells were fixed, permeabilized, and dyed following the manufacturer’s instructions (C0071S, Beyotime). Images were then visualized using an inverted fluorescence microscope (Nikon ECLIPS Ti2).

### 2.8 Isolation of leukocytes from tissues

Tumor-infiltrating leukocytes were obtained from fresh tissue samples as described previously (20). Briefly, fresh biopsy specimens from HCC patients were cut into small pieces and digested in RPMI 1640 medium supplemented with 0.002% DNase I (Roche), 0.05% collagenase IV (Sigma-Aldrich) and 10% FBS for 45 minutes at 37°C. The dissociated cells were passed through a 70-μm cell strainer and then erythrocytes were lysed and removed. The remaining cells were thoroughly rinsed and resuspended in PBS supplemented with 1% FBS (Gibco) for flow cytometry analysis.

### 2.9 Flow cytometry

Flow cytometry was performed as previously described (20). Before antibody staining, the cells were incubated with the Zombie Fixable Viability reagent for 15 minutes at room temperature. For surface staining, the cells were stained with fluorochrome-conjugated Abs for 30 minutes. For intracellular staining, the cells were fixed using the Fix/Perm solution (eBioscience), washed with the Perm/Wash buffer (eBioscience), and then incubated with fluorochrome-conjugated Abs for 30 minutes. For GPER1 staining, cells were incubated with GPER1 antibody before being stained with Alexa Flour 488-conjugated anti-rabbit IgG. Data was acquired using Cytoflex flow cytometer (Beckman Coulter) and analyzed using CytExpert software. Representative plots were created using Flowjo software 10 (Tree Star). The reagents and Abs used for flow cytometry are listed as follows: Zombie NIR™ (423105, Biolegend), Zombie Violet™ (423113, Biolegend); anti-human Abs including CD45-PE (304008, Biolegend), CD14-AF700 (557923, Biolegend), Ki67-PE (556027, Biolegend), Ki67-APC (350514, Biolegend), PD-L1-PC7 (558017, Biolegend) and GPER1 (PA5-109319, Thermo Fisher Scientific); anti-mouse Abs including CD45-BV605 (103140, Biolegend), CD11b-FITC (101206, Biolegend), Ly-6G-ECD (562700, BD Biosciences), F4/80-PE (123110, Biolegend), PD-L1-PC7 (124314, Biolegend) and Ki67-APC (652405, Biolegend).

### 2.10 Cell Counting Kit-8 assay

Human monocytes were seeded in 96-well plates with a density of 12,500 cells per well, and cultured in DMEM supplemented with 10% human AB serum for 7 days. Then the monocyte-derived macrophages were either left untreated or treated with 20% Huh7-TSN for 48 hours, in the presence or absence of G-1 (1 μM). Afterward, the cells were incubated with the CCK-8 (CK-04, KYD bio) solution for 2 hours, and absorbance was measured at 450 nm.

### 2.11 Quantitative real-time PCR

Total RNA was extracted using TRIzol reagent, and then 1μg RNA was used to synthesize cDNA with Color Reverse Transcription Kit (A0010CGQ, EZBioscience). Sequences of the primers used are listed as follows: *ESR1* (ERα), Forward: 5′- GCTTACTGACCAACCTGGCAGA -3′, Reverse: 5′- GGATCTCTAGCCAGGCACATTC -3′; *ESR2* (ERβ), Forward: 5′- AGAGTCCCTGGTGTGAAGCAAG, Reverse: 5′- GACAGCGCAGAAGTGAGCATC-3′; *GPER1*, Forward: 5′- TCTAAACTGCGGTCAGATGTGGC-3′, Reverse: 5′- TGTGAGGAGTGCAAGGTGACCAG-3′; *AR*, Forward: 5′- GACGACCAGATGGCTGTCATT-3′, Reverse: 5′- GGGCGAAGTAGAGCATCCT-3′. QPCR was performed in triplicate according to a standard protocol using Color SYBR Green qPCR Master Mix (A0012-R2, EZBioscience) with the LightCycler 480 System (Roche). To determine the levels of the analyzed RNA, their expression was normalized relative to human *GAPDH*.

### 2.12 Immunoblotting

human monocyte-derived macrophages were either left untreated or treated with 20% Huh7-TSN for 48 hours, in the presence or absence of G-1(1 μM). Then the protein was extracted for immunoblotting analysis. Immunoblotting was performed as described previously (14). Primary Abs used are listed as follows: anti-human Cyclin E1 (4129T, CST), Cyclin D1 (2978T, CST), CDK2 (2546T, CST), CDK4 (12790T, CST), p-AKT (13038S, CST), AKT (4685S, CST), p-Erk1/2 (4370T, CST), Erk1/2 (4695T, CST), β-actin (4970S, CST). HRP-linked anti-rabbit/mouse IgG Abs were purchased from CST.

### 2.13 Enzyme-Linked Immunosorbent Assay

Fresh biopsy specimens from HCC patients were weighed and fully ground to generate tissue homogenate. The cells were then lysed completely using an ultrasonic crusher, followed by centrifugation to obtain clarified supernatant. Methanol was added to the supernatant, and the mixture was incubated at room temperature for 10 minutes. After centrifugation, the supernatant was transferred to a clean tube and evaporated to dryness using centrifugal concentration drying system (Eppendorf). Assay buffer was added to reconstitute the precipitate, and then the estradiol content was immediately measured using an ELISA kit (501890, Cayman), following the manufacturer’s instructions.

### 2.14 Estimating immune cell scores of HCC tumor samples

The gene transcription expression data of HCC was obtained from The Cancer Genome Atlas (TCGA) database (22). Then the data was used to estimate the accumulation levels of various immune cells in 371 HCC tumor samples. This was achieved using the “IOBR” R package, employing the “Estimating the Proportion of Immune and Cancer cells” (“EPIC”) and the “xCell” methods (23).

### 2.15 Statistics

The statistical tests used are indicated in the figure legends. Two-tailed Student’s t-test or one-way ANOVA with Tukey’s multiple comparisons test was used to compare the means of two or multiple groups, respectively. Survival curves were calculated using the Kaplan-Meier method and analyzed with the log-rank test. The statistical analyses mentioned above were performed using GraphPad Prism 6. Univariate and multivariate analyses were performed using the Cox proportional hazards model (SPSS Statistics 21, IBM). *P* < 0.05 was considered statistically significant for all tests.

## 3 Results

### 3.1 Sex disparities in the accumulation and proliferation of macrophages in HCC tumors

**Figure 1:**
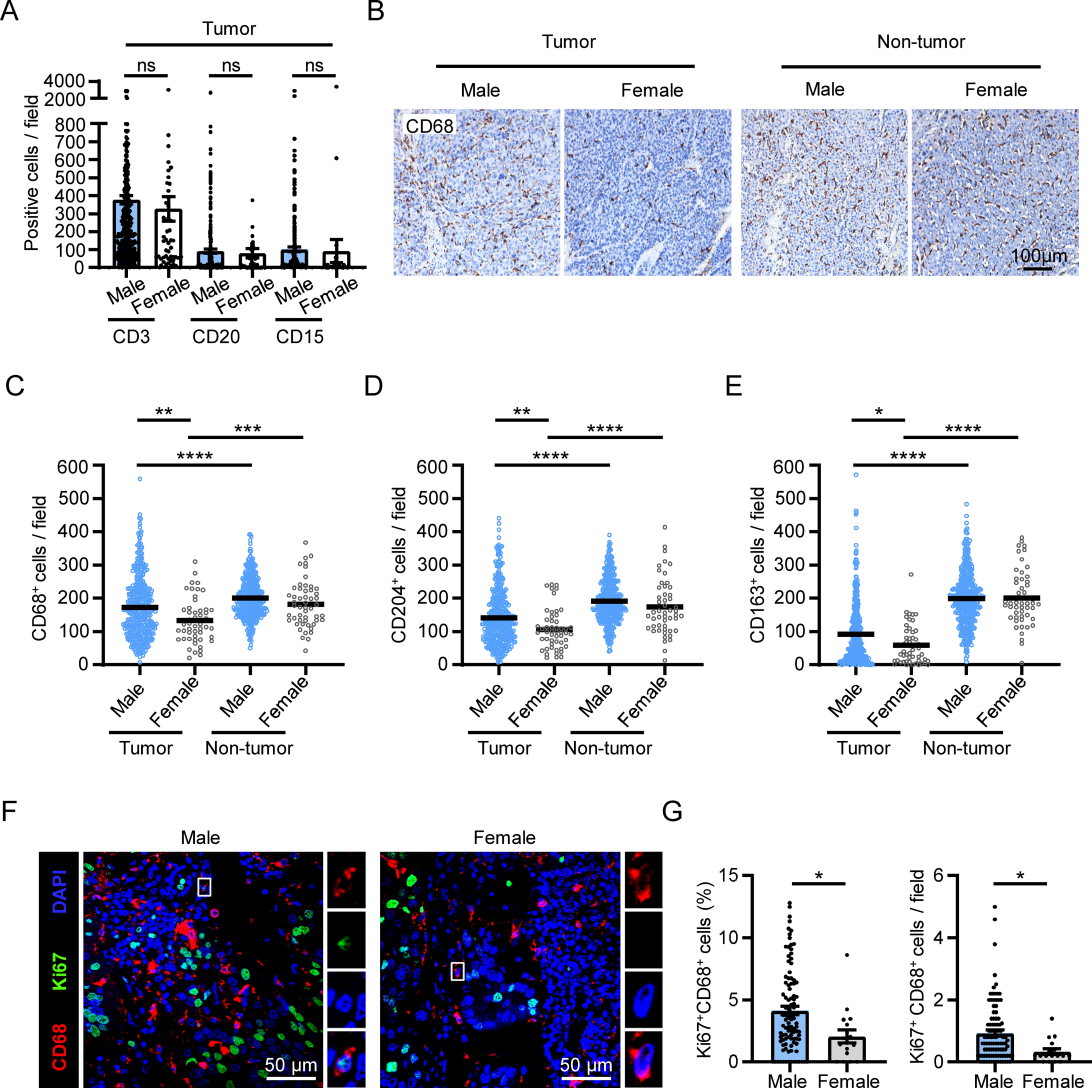
Sex disparities in the accumulation and proliferation of macrophages in HCC tumors. **(A–E)** IHC staining was performed on paraffin-embedded tissues from HCC patients (male, n = 381; female, n = 52). Statistical analysis of CD3, CD20, CD15 staining in the tumor tissue of HCC patients was shown in **(A)**. A representative CD68 staining is shown in **(B)**. The scale bar is 100 μm. **(F, G)** Fluorescence visualization and quantification of proliferating macrophages (Ki67^+^CD68^+^) among the total CD68^+^ macrophages in the tumor tissues of HCC patients (male, n = 100; female, n = 17). The scale bar is 50 μm. The results shown are represented as mean ± standard error of the mean (SEM), *p* values were obtained using nonpaired two-tailed Student’s t test. *p < 0.05, **p < 0.01, ***p < 0.001, ****p < 0.0001; ns, not significant.

To investigate the sex-related differences in the immune microenvironment of HCC tissues, we compared the density of various immune cells, determined by IHC, in tumor tissues derived from male and female patients in our previous cohort (24). While there were no significant differences in the density of CD3^+^ T cells, CD20^+^ B cells or CD15^+^ neutrophils between male and female patients, we observed a higher accumulation of CD68^+^ macrophages in the tumor tissues of male patients compared to female patients (**Figures 1A–C**; n _male_ = 381; n _female_ = 52). We also found higher levels of the markers CD204 and CD163, which are associated with a pro-tumor phenotype of macrophages in HCC (25, 26), in the tumor tissues of male patients compared to female patients (**Figures 1D, E**). These differences were not observed in the non-tumor regions. Additionally, we examined the immune cell infiltrations in HCC tumor samples from TCGA dataset using the “xCell” and the “EPIC” methods, and found that the level of macrophages, particularly M2-like macrophages, also showed the same sex discrepancy (**Supplementary Figures S1A, B**; n _male_ = 250; n _female_ = 121).

Self-replicating macrophages are enriched in HCC tumors and serve as an important mechanism for macrophage accumulation (14). Therefore, we set out to investigate whether sex difference was involved in the variation in macrophage self-replication. We conducted double immunofluorescence staining of Ki67 and CD68 in HCC tumor tissues from our previous cohort (14), and compared the level of Ki67^+^CD68^+^ cells between male and female patients (**Figure 1F**). The results revealed that both the percentage and the number of proliferating macrophages in tumor tissues were significantly higher in male patients compared to female patients (**Figure 1G**).

These results collectively reveal that there are sex disparities in macrophage proliferation and accumulation in HCC tumor tissues.

### 3.2 GPER1 expression negatively correlates with macrophage proliferation in HCC tumors

**Figure 2:**
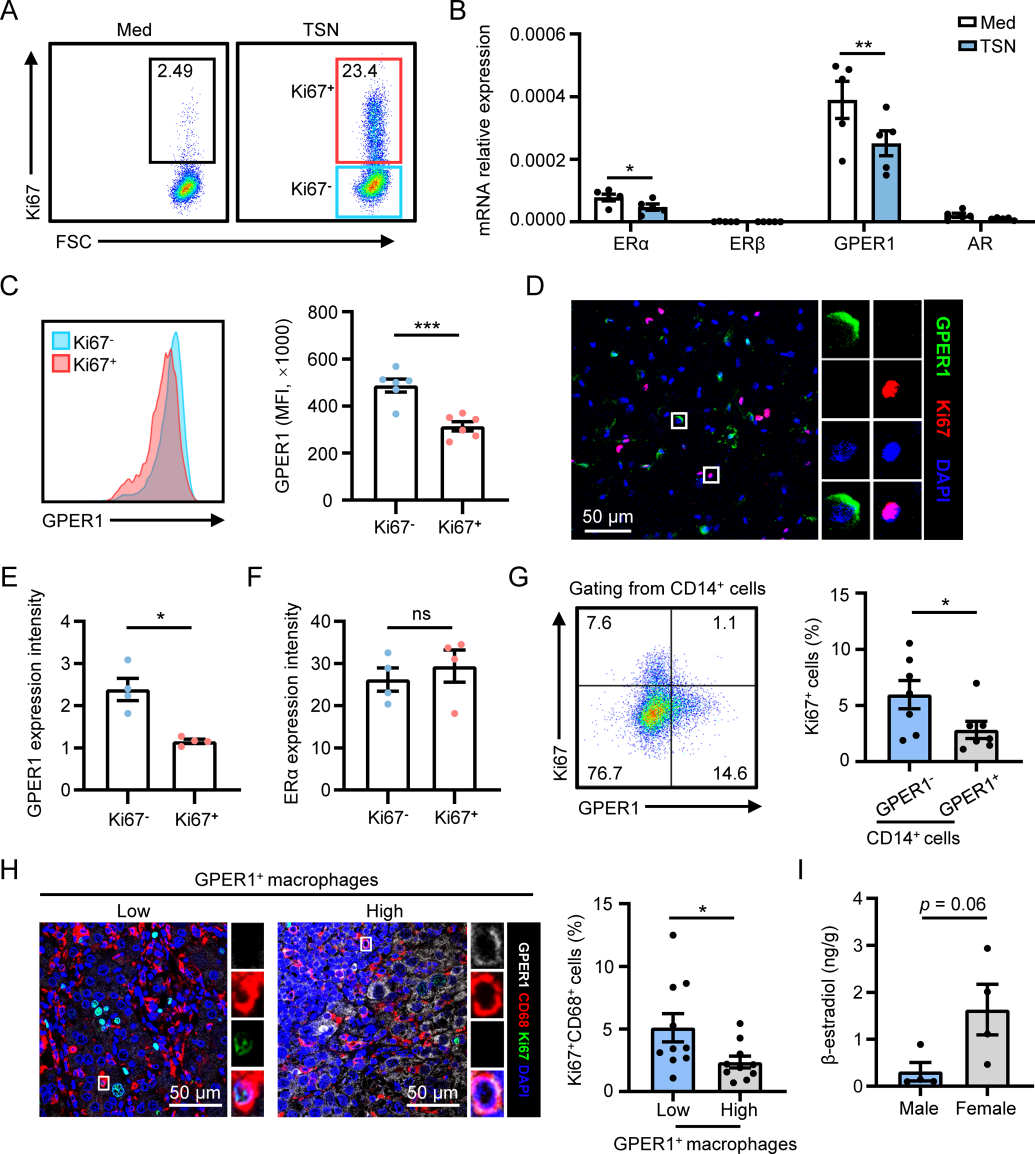
GPER1 expression negatively correlates with macrophage proliferation in HCC tumors. **(A–F)** Human monocyte-derived macrophages were treated with 20% culture supernatant from SK-Hep-1 cells (TSN) or control media (Med) for 48 hours. The proliferation level of macrophages was detected using flow cytometry analysis, and Ki67^-^ and Ki67^+^ macrophages were gated **(A)**. The expression levels of sex hormone receptors were examined using qPCR **(B**, n = 5). The expression levels of GPER1 in Ki67^-^ and Ki67^+^ macrophages gated in **(A)** were determined using flow cytometry **(C**, n = 6). The expression of Ki67, GPER1 and ERα in TSN-treated macrophages was visualized using confocal microscopy. Then the fluorescence intensity of GPER1 or ERα staining was compared between Ki67^-^ and Ki67^+^ macrophages **(D-F**, n = 4). The scale bar is 50 μm. **(G)** Representative dot plot and statistical analysis of the proliferation level of GPER1^-^ or GPER1^+^ macrophages isolated from fresh tumor tissues of HCC patients (n = 7). **(H)** HCC tumor samples were stained with anti-human CD68, GPER1 and Ki67 antibodies, and were then analyzed using confocal microscopy. The scale bar is 50 μm. Patients were divided into two groups according to the median frequency of GPER1^+^ macrophages among the total CD68^+^ macrophages, and the percentage of Ki67^+^ macrophages among the total CD68^+^ macrophages was compared between the two groups (n = 20). **(I)** 17β-estradiol concentrations (ng/g tissue) in HCC tumor tissues were examined using Enzyme-Linked Immunosorbent Assay (ELISA) (male, n = 4; female, n = 4). The results shown are represented as mean ± SEM. *P* values were obtained using paired or nonpaired two-tailed Student’s t test. *p < 0.05, **p < 0.01, ***p < 0.001; ns, not significant.

To investigate the mechanism behind this phenomenon, we established an *in vitro* model by incubating human monocyte-derived macrophages with tumor culture supernatants (TSN) to induce macrophage proliferation, as previously described (14) (**Figure 2A**). Then, qPCR was utilized to examine the expression of sex hormone receptors, specifically estrogen receptor α (ERα), estrogen receptor β (ERβ), GPER1 and androgen receptor (AR), which are known to play roles in sex-related differences in physiological and pathological conditions. The results showed that both GPER1 and ERα were significantly downregulated by TSN, whereas ERβ and AR were not affected (**Figure 2B**). Next, we compared the protein-level expression of GPER1 or ERα between proliferating and non-proliferating macrophages. Flow cytometry and immunofluorescence staining revealed that proliferating macrophages exhibited significantly lower expression of GPER1 when compared to non-proliferating cells in the presence of TSN (**Figures 2C–E**). However, no significant difference was observed in the expression of ERα between proliferating and non-proliferating macrophages (**Figure 2F** and **Supplementary Figure S2**).

The association between GPER1 expression and macrophage proliferation was examined in human HCC tumor tissues. Flow cytometry analysis of fresh tumoral leukocytes isolated from HCC patients showed that the proliferation level was significantly lower in CD14^+^GPER1^+^ cells compared to CD14^+^GPER1^-^ cells (**Figure 2G**). GPER1 expression in macrophages was also detected *in situ* using confocal microscopy. As shown in **Figure 2H**, the GPER1 positive signal was less prominent in Ki67^+^ macrophages compared to Ki67^-^ macrophages. Statistical analysis revealed that patients with a higher frequency of GPER1^+^ macrophages exhibited a significantly lower level of macrophage proliferation in tumor tissues. Additionally, the levels of 17β-estradiol (E2), the primary natural ligand for GPER1 (27, 28), were measured in HCC tumor tissues, and the result indicated that tumor tissues from female patients tended to exhibit a higher level of E2 compared to those from male patients (**Figure 2I**). Therefore, there is a negative correlation between the expression of GPER1 in macrophages and their proliferation, both *in vitro* and in HCC tumor tissues.

### 3.3 GPER1 activation restricts macrophage proliferation

**Figure 3:**
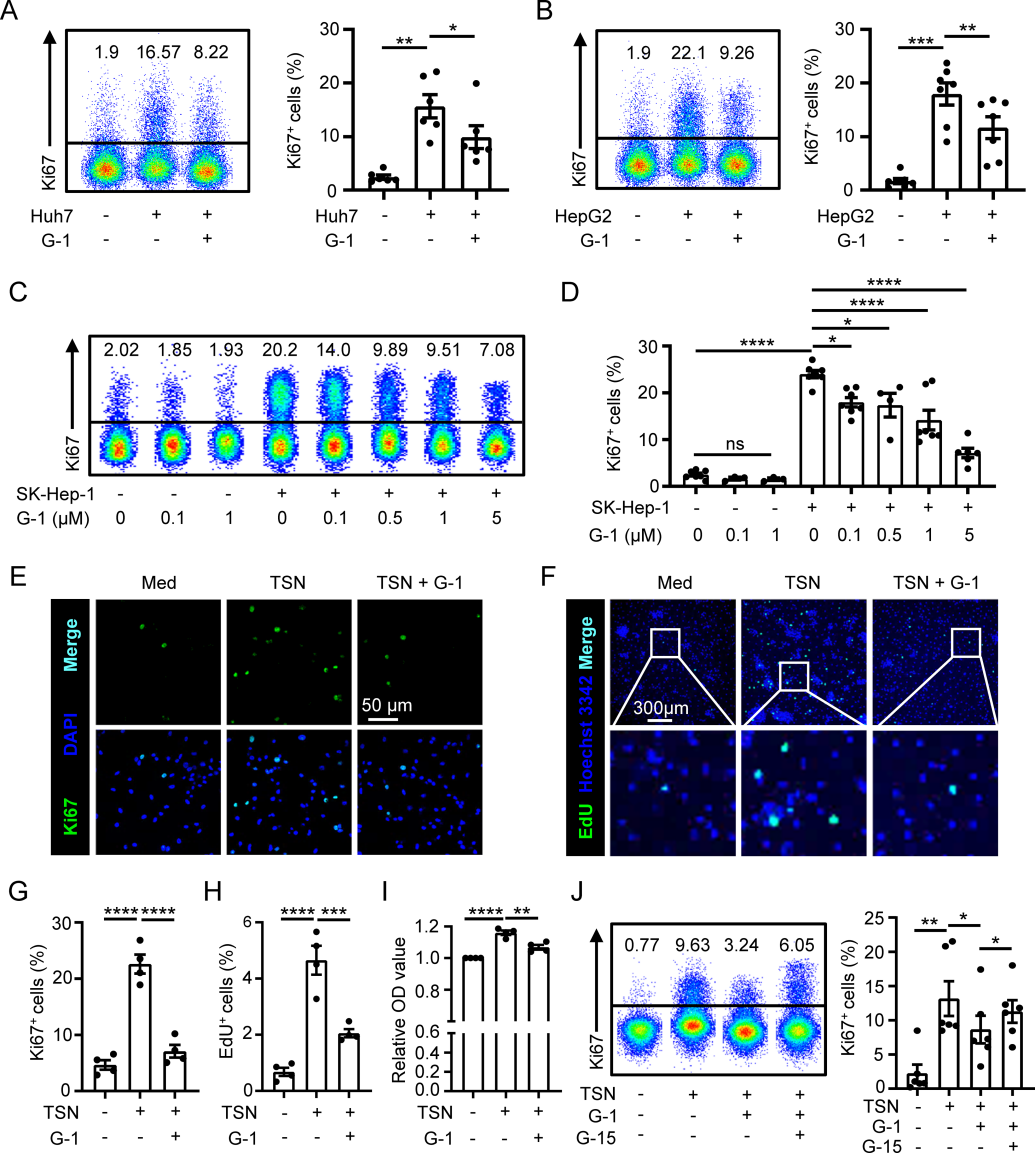
GPER1 activation restricts macrophage proliferation. **(A–D)** Human monocyte-derived macrophages were either untreated or treated with 20% TSN from Huh7, HepG2 or SK-Hep-1 cells for 48 hours, in the presence or absence of indicated concentrations of G-1 (1 μM for **A** and **B**). The percentages of Ki67^+^ macrophages were assessed using flow cytometry (n = 7). **(E–I)** Human monocyte-derived macrophages were either untreated (Med) or treated with 20% Huh7-TSN for 48 hours, in the presence or absence of G-1 (1 μM). Ki67^+^ **(E)** and EdU^+^ **(F)** macrophages were visualized using confocal microscopy. The scale bar is 50 μm in **(E)** and 300 μm in **(F)**. The statistical analysis of the percentages of Ki67^+^ (**G**, n = 4) and EdU^+^ (**H**, n = 4) macrophages is shown. Densities of macrophages were determined using a CCK-8 assay, and the optical density (OD) values of each donor were normalized relative to the corresponding value of the group without TSN treatment and then compared among different groups (**I**, n = 4). **(J)** Human monocyte-derived macrophages were either untreated or treated with Huh7-TSN for 48 hours, in the presence or absence of G-1 (1 μM) or G15 (0.1 μM). Ki67^+^ macrophages were assessed using flow cytometry (n = 6). The results shown are represented as mean ± SEM. *P* values were obtained using one-way ANOVA with Tukey’s multiple comparisons test. *p < 0.05, **p < 0.01, ***p < 0.001, ****p < 0.0001; ns, not significant.

Next, we set out to investigate the impact of GPER1 signaling on macrophage proliferation using G-1, a GPER1 specific agonist (29). The results showed that G-1 significantly suppressed the proliferation of macrophages induced by TSN from various hepatoma cell lines (**Figures 3A–D**). Moreover, the inhibitory effect of G-1 on TSN-induced macrophage proliferation was observed to be dose-dependent (**Figures 3C, D**). Immunofluorescence staining confirmed that G-1 treatment resulted in a notable decrease in the percentage of Ki67^+^ macrophages (**Figures 3E, G**). To determine DNA synthesis in macrophages, we conducted an EdU incorporation assay, which revealed an elevated frequency of EdU^+^ cells in macrophages exposed to TSN. However, this frequency was markedly reduced by the treatment of G-1 (**Figures 3F, H**). Additionally, the impact of GPER1 signaling on macrophage accumulation was examined using a CCK-8 assay. The results indicated that TSN increased the number of macrophages, while G-1 significantly decreased it (**Figure 3I**).

We further used a GPER1 specific antagonist G-15 to inhibit GPER1 signaling (30) that was activated by G-1. The results displayed that the suppression of macrophage proliferation mediated by G-1 was attenuated by G-15 treatment (**Figure 3J** and **Supplementary Figure S3**). In addition, we also examined the effect of E2 on macrophage proliferation, and the results showed that E2 inhibited TSN-induced macrophage proliferation, which was rescued by G-15 (**Supplementary Figure S4**).

Taken together, these data suggest that activation of GPER1 signaling restricts macrophage proliferation.

### 3.4 GPER1 signaling restrains macrophage proliferation by inhibiting the MEK/ERK/cyclin pathway

**Figure 4:**
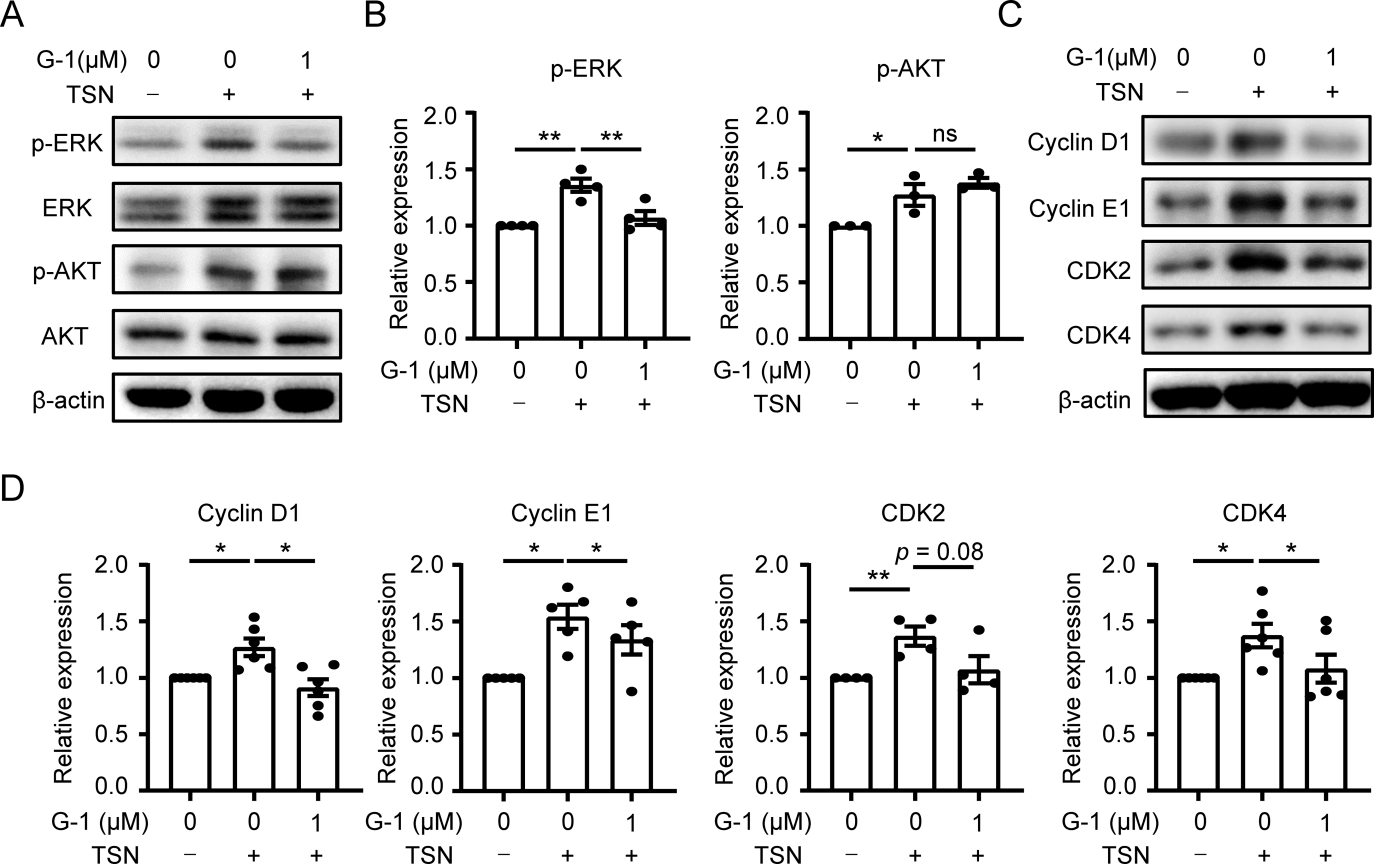
GPER1 signaling restrains macrophage proliferation by inhibiting the MEK/ERK/cyclin pathway. **(A–D)** Human monocyte-derived macrophages were either untreated or treated with 20% Huh7-TSN for 48 hours, in the presence or absence of G-1(1 μM). The levels of p-ERK, ERK, p-Akt, Akt, cyclin D1, cyclin E1, CDK2 and CDK4 were determined using immunoblotting **(A, C)**. Quantitative analysis of protein expression levels, normalized to β-actin, was performed and plotted (**B, D**, n = 4 or 6). The results shown in **(B, D)** are represented as mean ± SEM. *P* values were obtained using one-way ANOVA with Tukey’s multiple comparisons test. *p < 0.05, **p < 0.01; ns, not significant.

The PI3K/AKT and MEK/ERK pathways play crucial roles in regulating macrophage proliferation (14). Therefore, we investigated whether these signaling pathways are involved in the GPER1-mediated downregulation of macrophage proliferation. As expected, the levels of p-ERK and p-AKT significantly increased in macrophages exposed to TSN. G-1 treatment reversed the level of p-ERK, while the level of p-AKT was not significantly affected (**Figures 4A, B**). This indicates that GPER1 activation restricts macrophage proliferation by inhibiting the MEK/ERK pathway.

The ERK-mediated cyclin-dependent pathway is involved in cell-cycle progression and proliferation (31). Considering the reduced activity of DNA synthesis in TSN-treated macrophages after GPER1 activation (**Figures 3F, H**), we examined the expression levels of cyclins and cyclin-dependent kinases that regulate the entry of the DNA synthesis phase. Immunoblotting results showed that G-1 treatment significantly decreased the TSN-induced upregulation of cyclin D1, cyclin E1 and CDK4 (**Figures 4C, D**). These data suggest that GPER1 activation may restrict macrophage proliferation by downregulating the MEK/ERK/cyclin signaling pathway.

### 3.5 G-1 treatment inhibits macrophage proliferation and accumulation in HCC mouse models

**Figure 5:**
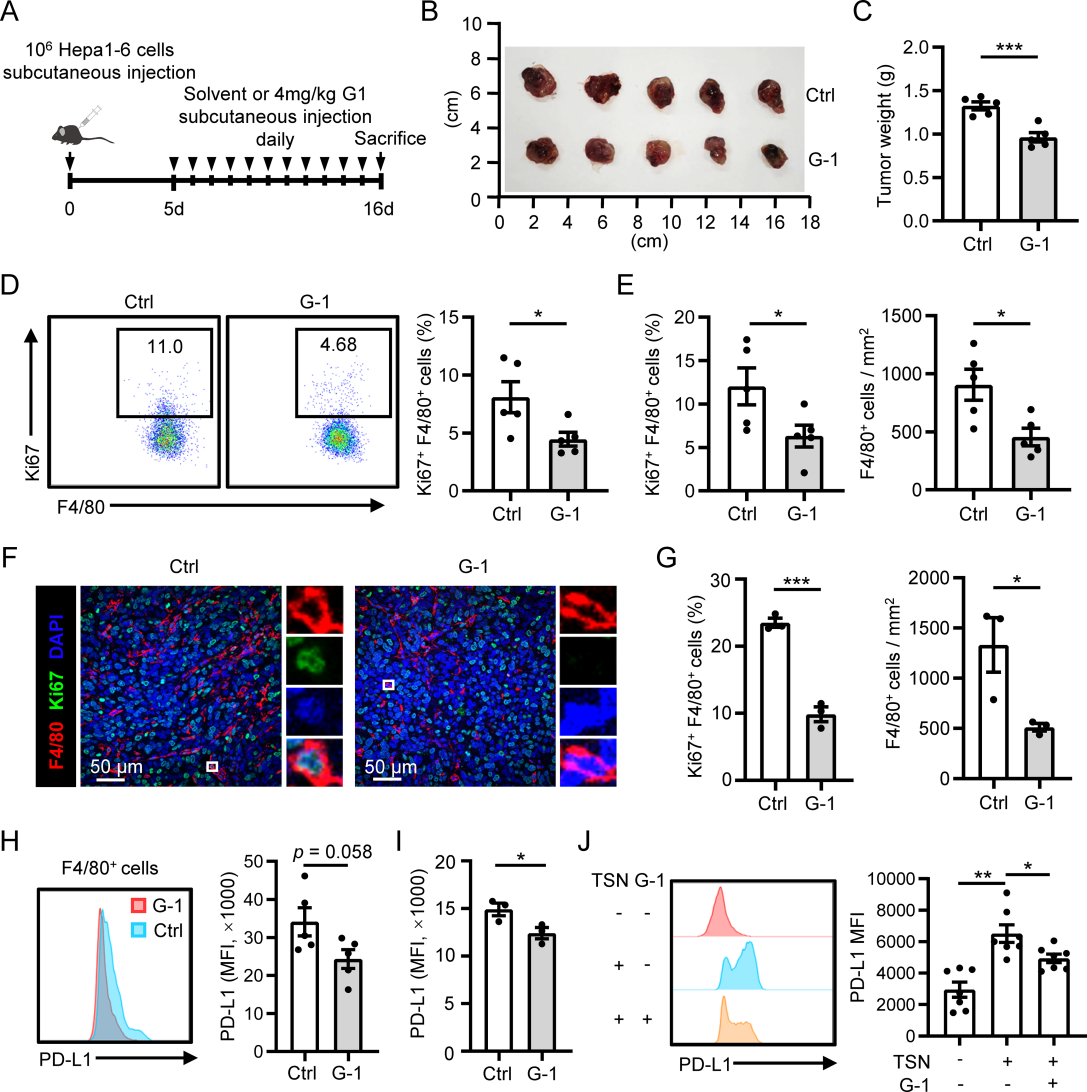
G-1 treatment inhibits macrophage proliferation and accumulation in HCC mouse models. **(A–F)** The subcutaneous tumor model was established (n = 5 for each group). Tumors were excised at the indicated time point, and their weights were analyzed **(B, C)**. Proliferation levels of F4/80^+^ macrophages in the tumor tissues were examined using flow cytometry **(D)**. **(E, F)** Immunofluorescence staining of F4/80 and Ki67 was performed on tumor sections. The proliferation level or density of F4/80^+^ macrophages was compared between the control and G-1 treated group. The scale bar is 50 μm. **(G)** The orthotopic tumor model was established (n = 3 for each group). Proliferation levels of F4/80^+^ macrophages in the tumor tissues were examined using flow cytometry (left). Densities of F4/80^+^ macrophages in the tumor tissues were analyzed using IHC (right). **(H, I)** PD-L1 expression on F4/80^+^ macrophages in tumor tissues were examined using flow cytometry and compared between the control and G-1 treated group in the subcutaneous tumor model **(H)** and orthotopic tumor model **(I)**, respectively. **(J)** Human monocytes were left untreated or treated with TSN in the presence or absence of G-1 for 7 days, and the expression of PD-L1 was examined using flow cytometry (n = 7). The results shown are represented as mean ± SEM. For **(C–I)**, *p* values are obtained by nonpaired two-tailed Student’s t test. One-way ANOVA with Tukey’s multiple comparisons test was used for **(J)**.

Next, we investigated whether the activation of GPER1 signaling with G-1 affects macrophage proliferation and accumulation in mouse models of HCC. We found that G-1 treatment significantly suppressed tumor growth in a subcutaneous Hepa1-6 tumor model (**Figures 5A–C**). Flow cytometry analysis displayed a significant reduction in macrophage proliferation in G-1-treated tumors (**Figure 5D**). Immunofluorescence staining of tumor tissues showed that G-1 treatment significantly decreased both the proliferation and the density of macrophages compared to the control group (**Figures 5E, F**). This inhibition of macrophage proliferation and accumulation by G-1 was also confirmed in an orthotopic Hepa1-6 tumor model (**Figure 5G**). Furthermore, we found that the macrophages in G-1-treated tumors tended to exhibit a lower level of PD-L1 in both the subcutaneous (**Figure 5H**) and the orthotopic HCC model (**Figure 5I**). The decrease in PD-L1 expression was also observed on human macrophages cultured *in vitro* when treated with G-1 (**Figure 5J**). Collectively, these results suggest that activating GPER1 signaling by G-1 restricts the proliferation and accumulation of macrophages in HCC mouse models.

### 3.6 GPER1 expression positively correlates with the survival of HCC patients

**Figure 6:**
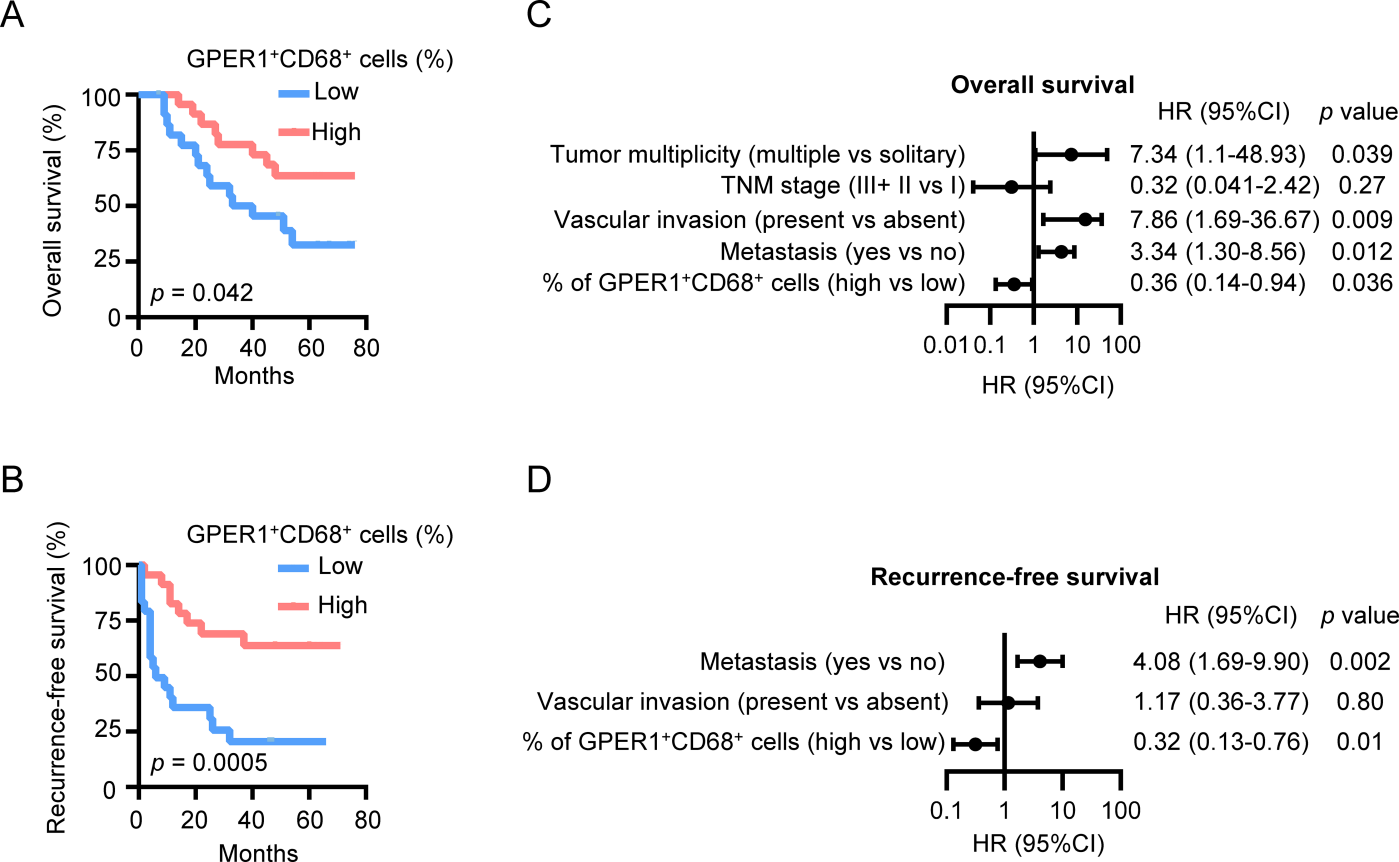
GPER1 expression positively correlates with the survival of HCC patients. **(A, B)** HCC patients were divided into two groups according to the median percentage of GPER1^+^ macrophages among the total macrophages in the tumor tissues. The cumulative overall survival **(A)** and recurrence-free survival **(B)** of patients were analyzed using the Kaplan-Meier method and compared by the log-rank test (n = 48). **(C, D)** Forest plot illustrating the associations between overall survival **(C)** or recurrence-free survival **(D)** and the clinical characteristics of HCC patients. A multivariate cox proportional hazards regression model was applied, incorporating variables that exhibited a significant univariate association with the outcome (*p* < 0.05). HR, hazard ratio; CI, confidence interval. *p < 0.05, **p < 0.01, ***p < 0.001.

To investigate the clinical significance of GPER1^+^ macrophages, percentages of GPER1^+^CD68^+^ cells in HCC tumor tissues were determined using immunofluorescence staining and confocal microscopy analysis. The patients were divided into two groups based on the median percentage of GPER1^+^CD68^+^ cells. As shown in **Figures 6A, B**, patients with a higher percentage of GPER1^+^ macrophages exhibited significantly longer OS and RFS compared to those with a lower level. Multivariate analysis indicated that the percentage of GPER1^+^ macrophages was an independent prognostic factor for both OS and RFS (**Figures 6C, D**, **Supplementary Table S2**). There was no obvious association between GPER1^+^ macrophages and the clinical characteristics of the patients (**Supplementary Table S3**).

These findings suggest that the presence of GPER1^+^ macrophages in HCC tumors predicts a better prognosis for HCC patients.

## 4 Discussion

The present study demonstrated the sex disparities in macrophage accumulation and proliferation in HCC tumor tissues. We found that GPER1, a 7-transmembrane G protein-coupled estrogen receptor, exhibited lower expression in self-replicating macrophages. When GPER1 signaling was activated through G-1 treatment, macrophage proliferation and accumulation were significantly suppressed in murine HCC tumors. Furthermore, patients with a higher percentage of GPER1^+^ macrophages exhibited longer OS and RFS compared to those with a lower level. These findings highlight the significant role of intrinsic sex hormone receptor signaling within macrophages in regulating macrophage proliferation and accumulation in HCC.

Sex-related differences in the tumor immune microenvironment have been observed in various cancers and been considered as potential explanations for the disparities in cancer incidence, prognosis, and response to treatments between sexes (4). For instance, the androgens/AR signaling has been found to suppress T cell immunity against cancer in males, resulting in faster tumor progression compared to females in colorectal cancer and melanoma (10, 11). On the other hand, the estrogens/ERα signaling has been shown to skew the polarization of macrophages towards an immune-suppressive state, leading to sex-specific differences in response to immune checkpoint inhibitors (ICIs) in melanoma (9). However, the sex-related mechanisms in determining the composition and function of the tumor microenvironment of HCC remain elusive. Macrophages, which are a major immune component in tumor tissues, have been widely reported to promote the progression of various types of cancer, including HCC (32–34). In this study, we demonstrated a higher accumulation of macrophages in tumors from male patients in two independent HCC cohorts. We observed that the accumulation of macrophages in tumors could be reduced by selectively targeting the GPER1 signaling with G-1 in mouse models of HCC, suggesting a potential strategy to modulate the macrophage pool by targeting estrogen receptor signaling.

Multiple environmental cues and intrinsic pathways influence the accumulation of macrophages in tumor tissues (35). We and other groups have demonstrated that macrophage proliferation within tumor tissues is a significant characteristic of malignancy (14, 36, 37) and an independent prognostic factor for poor survival of HCC patients (14). Therefore, our current study focuses on the proliferation of tumor-associated macrophages. We found that macrophages from male HCC patients displayed a higher level of proliferation compared to those from females. It should be noted that chemotaxis is also a key factor in the accumulation of monocytes/macrophages in tissues. The regulation of chemokines has been discussed in several studies, such as the CCL2/CCR2 axis, which promotes the trafficking of monocytes/macrophages into tumor tissues (38). Further exploration is warranted to determine whether sex disparities also contribute to these factors that influence the accumulation of macrophages in HCC tissues.

GPER1, a non-classical estrogen receptor, differs in structure, intracellular localization and functions from classical nuclear estrogen receptors (ERα, ERβ) (28). The estrogens/GPER1 signaling plays important roles in various physiological contexts and has been found to mediate sex differences in the incidence and severity of cardiovascular and autoimmune diseases (39–41). While the regulatory role of GPER1 in tumor cell proliferation has been explored in various cancers, including HCC (18, 19, 42), there has been less study on its effects on immune cell proliferation. In this study, we analyzed GPER1 expression in primary human macrophages *in vitro* and in HCC tumor tissues, and found lower levels of GPER1 in proliferating macrophages. Functional experiments demonstrated that macrophage proliferation was hindered by GPER1 specific agonist G-1. Moreover, we measured the levels of E2, the primary physiological ligand for GPER1, in the tumor tissues of HCC patients, and the results showed that E2 levels tend to be higher in female patients compared to males. Although the potential effects of G-1 treatment on tumor cells require further exploration, these findings may partially explain the aforementioned sex differences in macrophage proliferation and accumulation in HCC tumors, and highlight a novel role of GPER1 signaling in the regulation of the HCC microenvironment.

Several studies have shown that GPER1 interacts with other sex hormone receptors to regulate cell proliferation and function (43, 44). For instance, AR suppresses GPER1 signaling to promote cell proliferation in triple-negative breast cancer (43). Additionally, the balance between GPER1 and ERα plays a role in regulating vascular remodeling (44). We have observed that macrophages treated with TSN exhibited reduced expression of ERα which is another receptor for E2; however, no significant difference in ERα expression was found between proliferating and non-proliferating macrophages. It remains unclear whether this estrogen receptor can act antagonistically or synergistically with GPER1 to regulate macrophage function and phenotype.

In addition, we observed a decrease in PD-L1 expression on macrophages in the HCC mouse models treated with G-1. We also confirmed this effect in cultured human macrophages, suggesting that G-1 can modulate immune function. It has been reported that G-1 inhibits PD-L1 expression on the tumor cells of pancreatic ductal adenocarcinoma and melanoma, which enhances the efficacy of PD-1 targeted immunotherapy (45, 46). Clinical trials have recently been conducted on a GPER1 agonist called LNS8801, specifically for its use in combination therapies with ICIs in cancer treatment. These trials have demonstrated that LNS8801 exhibits a favorable safety profile when used alone or in combination with pembrolizumab (47, 48). Therefore, gaining a deep understanding of the influence of estrogen receptor signaling in modulating the tumor microenvironment may help in designing novel therapeutic strategies and selecting patients who may benefit from them.

In conclusion, our study provides insights into the role of intrinsic GPER1 signaling within macrophages in regulating their accumulation and function in the tumor microenvironment of HCC, and underscores the potential to develop tailored therapies for HCC treatment by considering the sex-related disparities among patients.

## Data Availability

The original contributions presented in the study are included in the article/**Supplementary Material**. Further inquiries can be directed to the corresponding author.
