# Supplementary material for: GPER1 signaling restricts macrophage proliferation and accumulation in human hepatocellular carcinoma
Source: Front Immunol. 2024 Nov 8;15:1481972. doi: 10.3389/fimmu.2024.1481972 (PMC11582010; doi:10.3389/fimmu.2024.1481972)
Supplement: Supplementary file 1 [file DataSheet1.pdf]

Figure S1

A

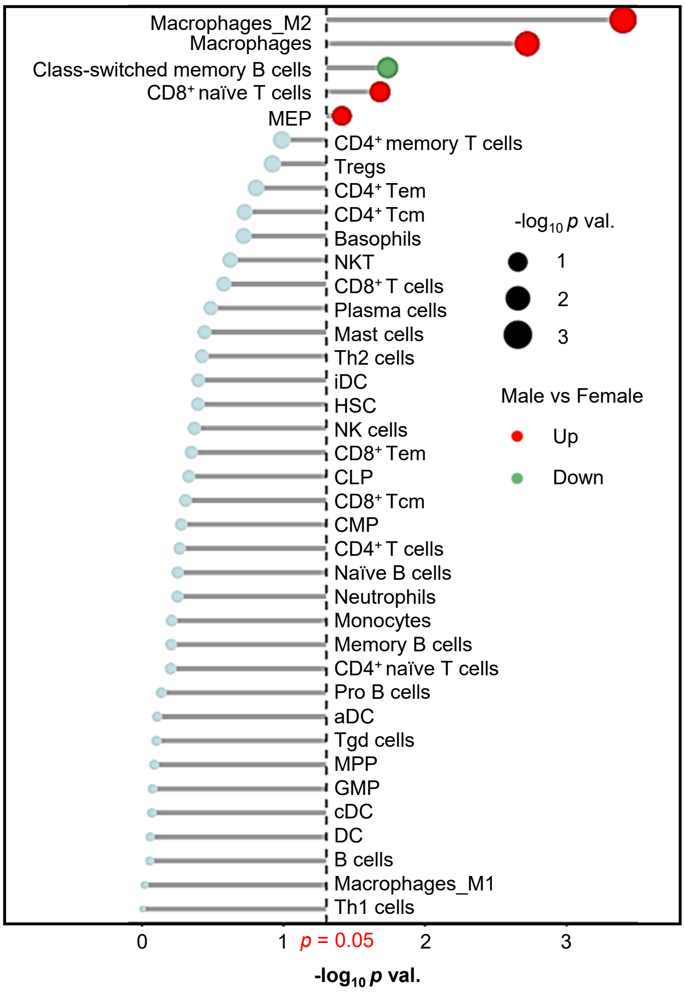

B

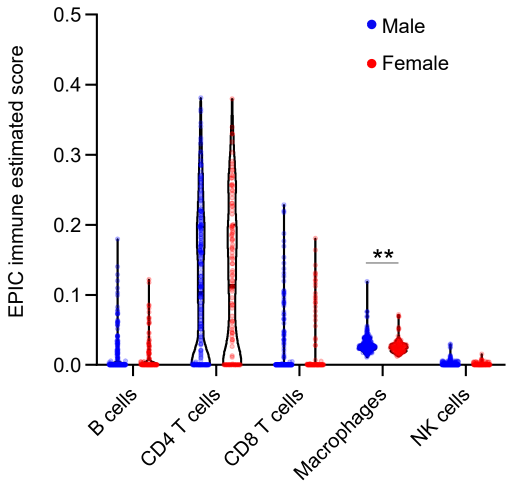

Fig. S1. Sex disparities in the accumulation of immune cells in HCC tumors. (**A** and **B**) The estimated scores of immune cells in HCC tumor tissues (male, n = 250; female, n = 121) were obtained by analyzing the transcriptomic data from TCGA database using the “xCell” (**A**) and “EPIC” (**B**) algorithms. Sex differences in the estimated scores of various immune cells were analyzed. *P* values were obtained by nonparametric Mann-Whitney test. $^{**}p < 0.01$.

Figure S2

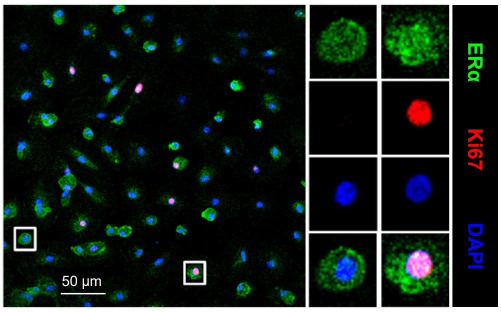

Fig. S2. The expression of ERα displays no significant difference between proliferating and non-proliferating macrophages. Human monocyte-derived macrophages were treated with 20% culture supernatant from SK-Hep-1 cells for 48 hours. The expression of Ki67 and ERα in macrophages was then visualized using confocal microscopy. The scale bar is 50 μm.

Figure S3

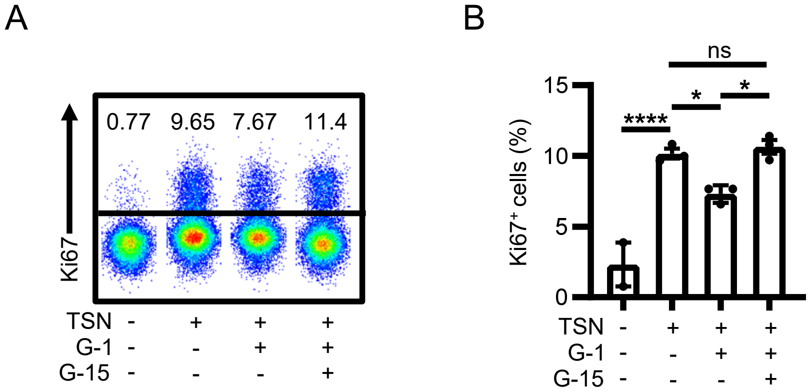

Fig. S3. GPER1 activation restricts macrophage proliferation. (**A** and **B**) Human monocyte-derived macrophages were untreated or treated with Huh7-TSN for 48 hours, in the presence or absence of G-1(0.1 μM) or G15(1 μM). Ki67$^+$ macrophages were assessed using flow cytometry (n = 3). The results shown are represented as mean ± SEM, and *p* values were obtained using one-way ANOVA with Tukey's multiple comparisons test. **p* < 0.05, *****p* < 0.0001; ns, not significant.

Figure S4

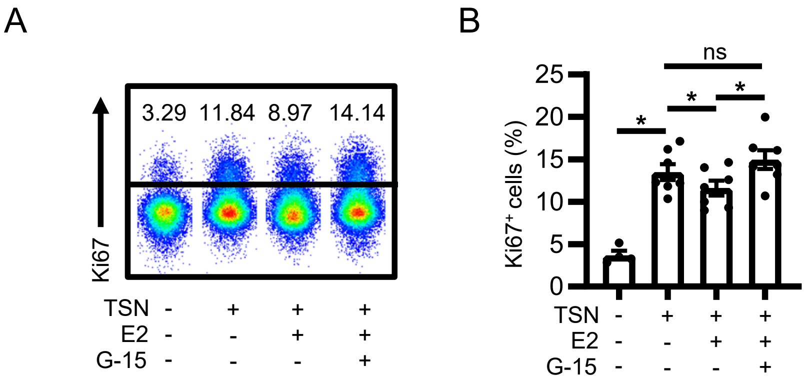

Fig. S4. E2 inhibits macrophage proliferation through activating GPER1. (**A** and **B**) Human monocyte-derived macrophages were untreated or treated with Huh7-TSN for 48 hours, in the presence or absence of E2 (50pM) or G15 (0.1μM). Ki67$^+$ macrophages were assessed using flow cytometry (n = 7). The results shown are represented as mean ± SEM, and *p* values were obtained using one-way ANOVA with Tukey's multiple comparisons test. **p* < 0.05, ns, not significant.

Table S1. Clinical characteristics of 48 patients with HCC

Variables	No. of patients
Case (n)	48
Age, years (≥ 50 vs <50)	26/22
Sex (male vs female)	38/10
HBsAg (positive vs negative) *	44/3
Cirrhosis (present vs absent)	34/14
ALT, U/L (≥ 40 vs < 40)	18/30
AFP, ng/ml (≥ 25 vs < 25)	35/13
Tumor size, cm (≥ 5 vs < 5)	34/14
Tumor multiplicity (multiple vs solitary)	16/32
Vascular invasion (present vs. absent)	5/43
Metastasis [A] (yes vs no)	18/30
TNM stage (II + III vs I)	20/28
Tumor differentiation (III + IV vs I + II)	22/26

Abbreviation: AFP, α-fetoprotein; ALT, alanine aminotransferase; HBsAg, hepatitis B surface antigen.

[A], patients who developed intrahepatic or extrahepatic metastases were considered as cases with metastasis.

*, information of indicated characteristics is not available for some patients.

Table S2. Univariate and multivariate analysis of factors associated with overall survival and recurrence-free survival of HCC patients

Variables	OS				RFS			
	Univariate *p*	Multivariate			Univariate *p*	Multivariate		
		HR	95% CI	*p*		HR	95% CI	*p*
Age, years (≥ 50 vs <50)	0.60			n.a.	0.12			n.a.
Sex (male vs female)	0.11			n.a.	0.37			n.a.
HBsAg (positive vs negative) *	0.52			n.a.	0.28			n.a.
Cirrhosis (present vs absent)	0.66			n.a.	0.12			n.a.
ALT, U/L (≥40 vs ≤40)	0.83			n.a.	0.36			n.a.
AFP, ng / ml (≥ 25 vs < 5)	0.21			n.a.	0.51			n.a.
Tumor size, cm (≥ 5 vs < 5)	0.31			n.a.	0.48			n.a.
Tumor multiplicity (multiple vs solitary)	**0.034**	7.34	1.1-48.93	**0.039**	0.086			n.a.
Vascular invasion (present vs. absent)	**< 0.0001**	7.86	1.69-36.67	**0.009**	**0.016**	1.17	0.36-3.77	0.80
Metastasis [A] (yes vs no)	**< 0.0001**	3.34	1.30-8.56	**0.012**	**< 0.0001**	4.08	1.69-9.90	**0.002**
TNM stage (II + III vs I)	**0.02**	0.32	0.041-2.42	0.27	0.068			n.a.
Tumor differentiation (III + IV vs I + II)	0.30			n.a.	0.16			n.a.
Intra-tumoral $GPER1^{+}CD68^{+}$ cells (high vs low)	**0.049**	0.36	0.14-0.94	**0.036**	**0.002**	0.32	0.13-0.76	**0.01**

Cox proportional hazards regression model. Variables used in multivariate analysis were adopted from univariate analysis. The bold terms represent statistical significance.

Abbreviation: CI, confidence interval; HR, hazard ratio; n.a., not adopted.

*, information of indicated characteristics is not available for some patients.

[A], patients who developed intrahepatic or extrahepatic metastases were considered as cases with metastasis.

Table S3. Associations of the intra-tumoral GPER1$^+$CD68$^+$ cells with clinical characteristics of HCC patients

Variables	% GPER1$^+$CD68$^+$ cells		p value #
	Low	High	
Age, years (≥ 50 vs <50)	12/12	14/10	0.56
Sex (male vs female)	17/7	21/3	0.16
HBsAg (positive vs negative) *	22/2	22/1	0.58
Cirrhosis (present vs absent)	15/9	19/5	0.20
ALT, U/L (≥40 vs ≤40)	10/14	8/16	0.55
AFP, ng/ml (≥ 25 vs < 25)	19/5	16/8	0.33
Tumor size, cm (≥ 5 vs < 5)	18/6	16/8	0.53
Tumor multiplicity (multiple vs solitary)	8/16	8/16	1
Vascular invasion (present vs. absent)	4/20	1/23	0.16
Metastasis [A] (yes vs no)	12/12	6/18	0.074
TNM stage (II + III vs I)	11/13	9/15	0.56
Tumor differentiation (III + IV vs I + II)	10/14	12/12	0.56

#, p value was analyzed by Chi-square test.

A, patients who developed intrahepatic or extrahepatic metastases were considered as cases with metastasis.

*, information of indicated characteristics is not available for some patients.
