# Supplementary figures and images for: GPER1 signaling restricts macrophage proliferation and accumulation in human hepatocellular carcinoma
Source: Front Immunol. 2024 Nov 8;15:1481972. doi: 10.3389/fimmu.2024.1481972 (PMC11582010; doi:10.3389/fimmu.2024.1481972)

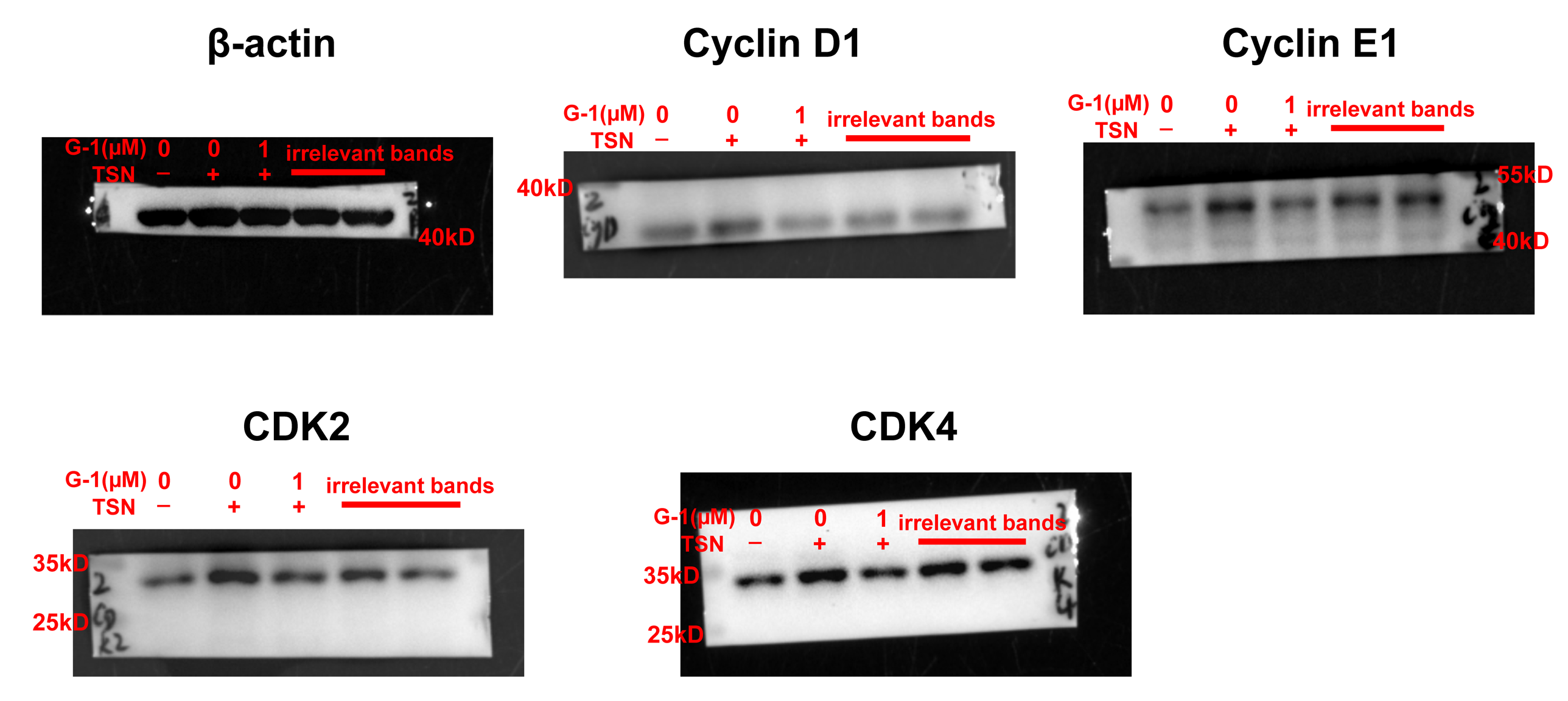

Supplement: Supplementary file 3 [file Image2.tif]
